# A conjugate of methotrexate and an analog of luteinizing hormone releasing hormone shows increased efficacy against prostate cancer

**DOI:** 10.1038/srep33894

**Published:** 2016-09-22

**Authors:** Shengsheng Zhu, Qinxia Wang, Juan Jiang, Yongwei Luo, Zuyue Sun

**Affiliations:** 1 Department of Pharmacology and Toxicology, Shanghai Institute of Planned Parenthood Research, Shanghai, 200032, PR China

## Abstract

LHRH receptor, is over-expressed in a variety of human tumors and, is a potential binding site for targeted metastatic prostate cancer therapy. The objectives of our study were to synthesize a bioconjugate of the LHRH analog [DLys^6^]-LHRH and the anti-tumor agent methotrexate and test the hypothesis that [DLys^6^]-LHRH-MTX targets and inhibits prostate cancer cell growth *in vitro* and *in vivo*. The results of *in vitro* studies, showed that both [DLys^6^]-LHRH-MTX and MTX displayed superior cytotoxicity against prostate cancer cells in a concentration-dependent manners, with IC_50_ concentrations for PC-3 cells of, 1.02 ± 0.18 μmol/L and 6.34 ± 1.01 μmol/L; for DU-145 cells, 1.53 ± 0.27 μmol/L and 8.03 ± 1.29 μmol/L; and for LNCaP cells, 1.93 ± 0.19 μmol/L and 9.68 ± 1.24 μmol/L, respectively. The IC_50_ values of [DLys^6^]-LHRH-MTX and MTX were 110.77 ± 15.31 μmol/L and 42.33 ± 7.25 μmol/L, respectively. Finally, [DLys^6^]-LHRH-MTX significantly improved the anti-tumor activity of MTX in nude mice bearing PC-3 tumor xenografts. The inhibition ratios of tumor volume and tumor weight in the [DLys^6^]-LHRH-MTX treated group were significantly higher than those in the MTX-treated group. Tumor volume doubling time was also significantly extended from 6.13 days in control animals to 9.67 days in mice treated with [DLys^6^]-LHRH-MTX. In conclusion, [DLys^6^]-LHRH -MTX may be useful in treating prostate cancer.

Prostate cancer is the most commonly diagnosed cancer in American men, with an estimated 238590 new cases of prostate cancer and 29720 deaths in 2013 alone^1^,^2^. In China, the incidence of prostate cancer is increasing annually and there were an estimated 236600 new cases of prostate cancer and 65900 deaths due to prostate cancer in 2014^3^. Prostate cancer is one of the leading causes of cancer-related deaths mainly owing to its metastasis to distant tissues such as the lung, liver, and bone^4^,^5^. Currently, androgen ablation is a commonly prescribed treatment option for localized disease but this treatment modality has a limited scope, especially for hormone-refractory prostate cancers^6^,^7^. Moreover, other treatment such as chemotherapy, radiation, surgery or a combination of these modalities are largely ineffective against advanced prostate cancer^5^,^8^,^9^,^10^,^11^. One of the major problems in cancer chemotherapy is the severely toxic side effects of anticancer drugs designed to destroy rapidly dividing cells, including those found in healthy tissues^12^. These severe side effects often result in dose reduction, treatment delay or discontinuation of therapy. To overcome these disadvantages, various systems have been developed to deliver the anti-cancer drugs used in chemotherapy using methods that decrease toxicity and limit nonspecific activity. Indeed, targeted drug delivery systems represent an advanced approach for the deliver of anti-cancer drugs. Cancer cell targeting can be achieved by adding several targeting moieties to the drug delivery system, such as receptor ligands, sugars, antibodies, hormones and hormone analogs, specifically directed to receptor binding sites on cancer cells^10^,^13^,^14^.

It is well known that that luteinizing hormone-releasing hormone (LHRH) and its receptor(LHRHR) are components of an autocrine/paracrine regulatory system of cell proliferation found in a number of human malignant tumors, including cancers of prostate, breast and ovary^15^,^16^,^17^. Available data strongly suggest that approximately 86% of human prostatic cancers, 50% of breast cancers and 80% of ovarian cancers express high-affinity binding sites for LHRH^15^,^18^. Although prostate cancers express LHRH receptors, and normal prostate cells do not, few findings have been published regarding the role of LHRH and LHRHR in prostate cancer. However, LHRH decapeptides have been used to deliver anti-cancer drugs specifically to cancer cells expressing their receptors. For example, Schally and Nagy^19^ used potent LHRH antagonists as targeting moieties for a variety of cancer drugs including doxorubicin (DOX). Studies on the conjugates [DLys^6^]-LHRH-DOX and [DLys^6^]-LHRH-2-pyrrolino-DOX showed increased efficacy of doxorubicin, as the LHRH analog maintained its highly targeted binding affinity while the drug retained its cytotoxic effects on the tumor cells^19^,^20^. Turner and Yates^21^ reported that conjugates of lytic peptides and LHRH are very effective in destroying prostate cancer xenografts that express LHRH receptors. Hansel *et al*. conjugated [DLys^6^]-LHRH and curcuimn and examined the efficacy of the conjugate ([DLys^6^]-LHRH–curcumin) against pancreatic cancer cells *in vitro* and *in vivo*, their results showed that the conjugate of [DLys^6^]-LHRH-curcumin was effective at treating pancreatic cancer^22^.

Methotrexate(MTX) was one of the first anti-metabolite drugs used in cancer therapy; this compound, can effectively deactivate the metabolism of diseased cells through programmed cell death and apoptosis^23^. To date, MTX continues to be used extensively in the treatment of various malignancies including childhood acute lymphocytic leukemia, osteosarcoma, non-Hodgkin’s lymphoma, Hodgkin’s disease, head and neck cancer, lung cancer, prostate cancer and breast cancer^24^. MTX ceases intracellular folate metabolism and finally blocks the synthesis of thymine and purines, leading to theimpairment of tumor growth and induction of cell death by the secondary genotoxic effects or apoptosis. Unfortunately, the development of multidrug resistance in cancer cells significantly restricts the effectiveness of this compound. Thus, the clinical application of MTX has remained limited^25^. To overcome this limitation, in the current study, we conjugated [DLys^6^]-LHRH and MTX. This article reports the preparation and characterization of [DLys^6^]-LHRH-MTX using nuclear magnetic resonance (NMR) spectroscopy, and liquid chromatography-mass spectrometry (LC-MS) analysis. Further, it reports the efficacy of the conjugate *in vitro* in prostate cancer cell lines and *in vivo* in a mouse PC-3 xenograft model.

## Results and Discussion

### [DLys^6^]-LHRH-MTX inhibits the proliferation of prostate cancer cells

**Figure 1:**
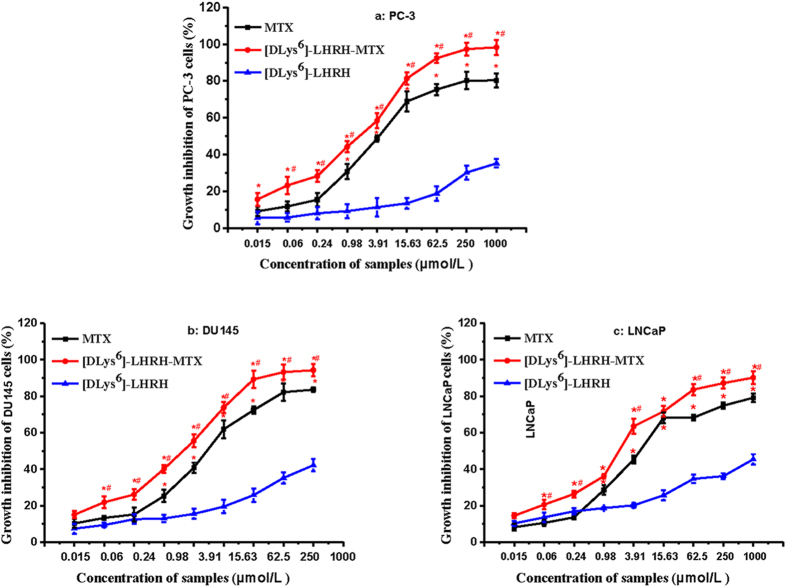
*In vitro* cytotoxicity of [DLys^6^]-LHRH-MTX, MTX and [DLys6]-LHRH against prostate cancer cells as determined by MTT assay. Untreated control cells corresponded to 100% viability. The data presented are the average of three independent experiments (n = 3, *p < 0.05 vs. [DLys^6^]-LHRH, ^#^p < 0.05 vs. MTX).

The growth inhibitions effects of [DLys^6^]-LHRH-MTX, MTX and [DLys^6^]-LHRH on prostate cancer cell lines were investigated at various concentrations after 24 h of treatment by MTT assay. [DLys^6^]-LHRH-MTX inhibited prostate cancer cell proliferation in a dose-dependent manner in three prostate cancer cell lines as shown in Fig. 1a–c. The IC_50_ for [DLys^6^]-LHRH-MTX varied for different prostate cancer cell lines: for PC-3, this value was 1.02 ± 0.08 μmol/L, for DU145, 1.53 ± 0.27 μmol/L; and for LNCaP 1.93 ± 0.19 μmol/L; The IC_50_ of MTX against the prostate cancer cell lines was as follows: PC-3, 6.34 ± 1.01 μmol/L, DU145, 8.03 ± 1.29 μmol/L; and LNCaP 9.68 ± 1.24 μmol/L; All of the IC_50_ values of the different compound are summarized in Table 1. As shown in Fig. 1 and Table 1, [DLys^6^]-LHRH-MTX exhibited stronger cytotoxicity (IC_50_ = 1.02 ± 0.18~1.93 ± 0.19 μmol/L) than free MTX (IC_50_ = 6.34 ± 1.01~9.68 ± 1.24 μmol/L). The IC_50_ values for prostate cancer cells (PC-3, DU145 and LNCaP) was 6.34 ± 1.01~9.68 ± 1.24 μmol/L for MTX alone, the conjugation of [DLys^6^]-LHRH lowered the IC_50_ concentrations to 1.02 ± 0.18~1.93 ± 0.19 μmol/L for [DLys^6^]-LHRH-MTX. These results suggest that conjugation did not hinder MTX activity. [DLys^6^]-LHRH showed no cytotoxicity against prostate cancer cell lines, which is consistent with reports showing that LHRH alone has minimal apoptotic effects on ovarian and endometrial cancers^26^.

**Figure 2:**
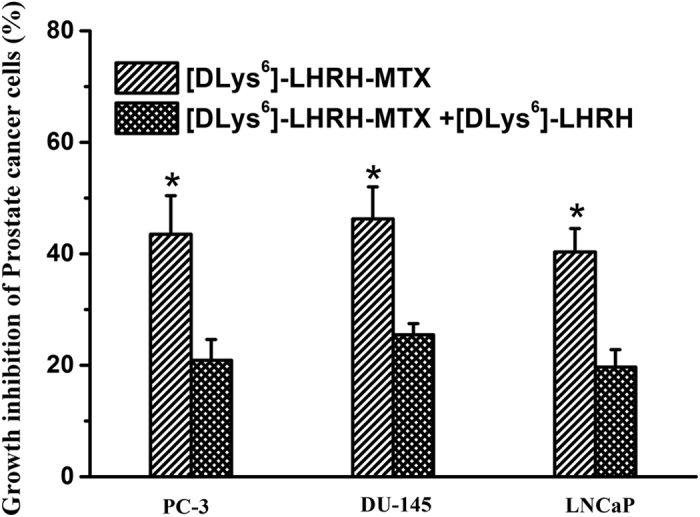
The effect of coincubation of [DLys^6^]-LHRH-MTX and free peptide on prostate cancer cells cytotoxicity. Prostate cancer cells (5000 cells/well) were coincubated with 0.98 μmol/L of [DLys^6^]-LHRH-MTX and 0.98 μmol/L of [DLys^6^]-LHRH for 24 h. The coincubation of [DLys^6^]-LHRH decreased the cytotoxicity of [DLys^6^]-LHRH-MTX. The data presented are the average of three independent experiments. (n = 3, *P < 0.05).

When prostate cancer cells were coincubated with [DLys^6^]-LHRH and [DLys^6^]-LHRH-MTX, the cytotoxic effect of [DLys^6^]-LHRH-MTX was significantly lower than that of [DLys^6^]-LHRH-MTX alone, indicating that [DLys^6^]-LHRH-MTX and [DLys^6^]-LHRH compete for binding sites on the prostate cancer cell membrane (Fig. 2)^26^.

**Figure 3:**
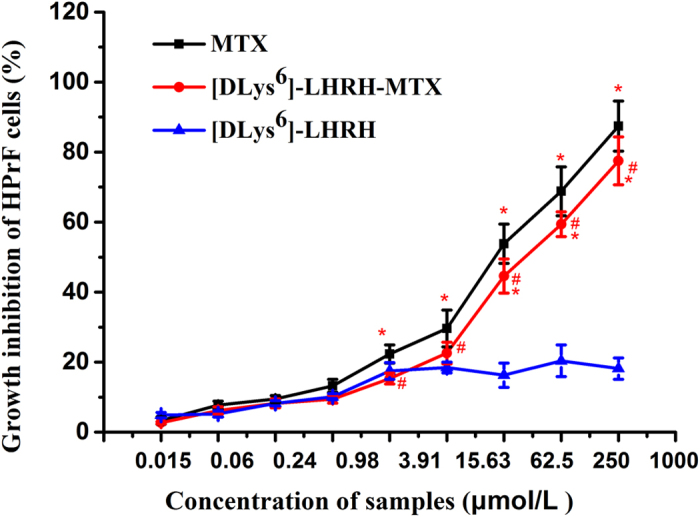
Cytotoxicity of MTX and [DLys^6^]-LHRH-MTX to normal primary HPrF cells. HPrF cells were exposed to MTX or [DLys^6^]-LHRH-MTX at 0.015-1000 μmol/L. Growth inhibition was determined by MTT assay. (n = 3, *p < 0.05 vs. [DLys^6^]-LHRH, ^#^p < 0.05 vs. MTX).

To determine the selectivity of LHRH-MTX, we performed a MTT experiment with normal human prostate fibroblast (HPrF) cells. The IC_50_ of [DLys^6^]-LHRH-MTX in HPrF cells was 110.77 ± 15.31 μmol/L, and the IC_50_ of MTX in HPrF cells was 42.33 ± 7.25 μmol/L. As shown in Table 1 and Fig. 3, both MTX and [DLys^6^]-LHRH-MTX showed cytotoxicity against HPrF cells while the [DLys^6^]-LHRH showed no cytotoxicity against HPrF cells. It is well known that the clinical disadvantages of MTX include its high dosage and dose-limitation. Together, our results indicate that [DLys^6^]-LHRH-MTX may improve the therapeutic efficacy of MTX^25^.

### [DLys^6^]-LHRH-MTX induces apoptosis in prostate cancer cells

**Figure 4:**
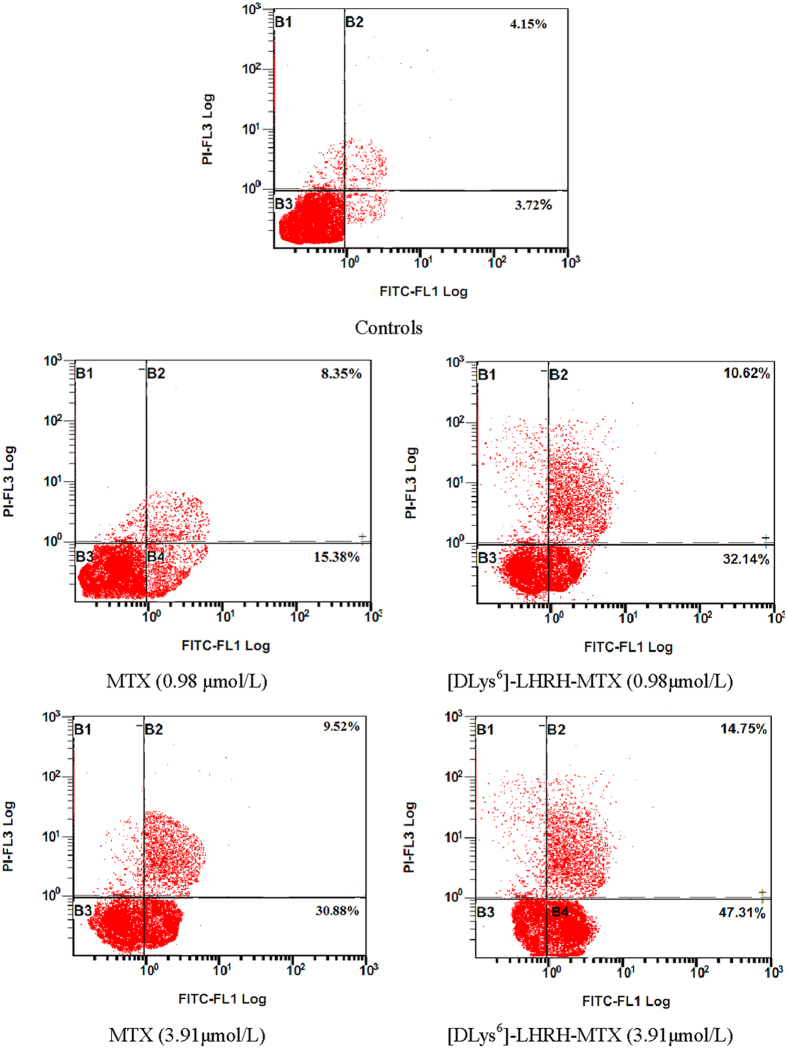
Quantitative apoptotic measurement in PC-3 cells after treatment with [DLys^6^]-LHRH-MTX and MTX, the concentration-dependent effects on apoptosis following treatment with MTX and [DLys^6^]-LHRH-MTX for 24 at doses of 0.98 μmol/L and 3.91 μmol/L were determined by flow cytometry analysis. Results were expressed as a dot plot of Annexin V -FITC vs. PI. The dot plot from the flow cytometry analysis reveals the four different populations of cells. Top left: cell debris (AV−/PI+); top right: necrotic/late apoptotic cells (AV+/PI+); bottom left: live cells (AV−/PI−); and bottom right: early apoptotic cells (AV−/PI+). The data listed in the first line correspond to the cells treated with 0.98 μmol/L and that in the second line correspond to the cells treated with 3.91 μmol/L.

To quantitatively investigate apoptotic and early necrotic events in these treated cultures, the fluorescent probe Annexin V-FITC and PI were used, The presence of a apoptotic and early necrotic cells was then determined and quantified by flow cytometry analysis which clearly differentiated normal cells with low Annexin V-FITC and low PI staining, apoptotic cells with high Annexin V-FITC and low PI staining, necrotic cells with high Annexin V-FITC and high PI staining and cell debris with low Annexin VFITC and high PI staining. Figure 4 shows that apoptotic cells were detected after treatment with MTX and [DLys^6^]-LHRH-MTX for 24 h although the percentage of apoptotic cells varied with each therapy. Cells treated with [DLys^6^]-LHRH-MTX at 0.98 μmol/L showed 32.14% early apoptotic (AV+/PI−) populations and 10.62% late apoptotic/early necrotic (AV+/PI+) cells, while for MTX treatment, these percentages were 15.38% and 8.35%. In addition, it should be noted that the apoptotic and necrotic cell death induced by treatment increased with the drug concentration up to 3.91 μmol/L, these percentages increased to 47.31% and 14.75% for [DLys^6^]-LHRH-MTX, and 30.88% and 9.52% for MTX, respectivetly. Figure 4 shows that greater apoptotic cell death was observed following treatment with [DLys^6^]-LHRH-MTX when compared to MTX alone. Conjugates of [DLys^6^]-LHRH have previously been shown to exhibit higher rates of apoptosis compared with their unconjugated counterparts^27^. The higher apoptosis observed with the [DLys^6^]-LHRH-MTX conjugate is likely due to enhanced uptake of MTX via the LHRHR^27^,^28^,^29^. Apparently, more early apoptotic cells (Annexin V-FITC+/PI−) than late apoptotic/early necrotic cells (Annexin V-FITC+/PI+) were observed in cells treated with the two compounds, suggesting progression from early apoptotic to late apoptosis/early necrosis. These results demonstrate that apoptotic cell pathways, but not directly necrotic one, seem to be activated by these compounds.

### Evaluation of *in vivo* anti-tumor activity

**Figure 5:**
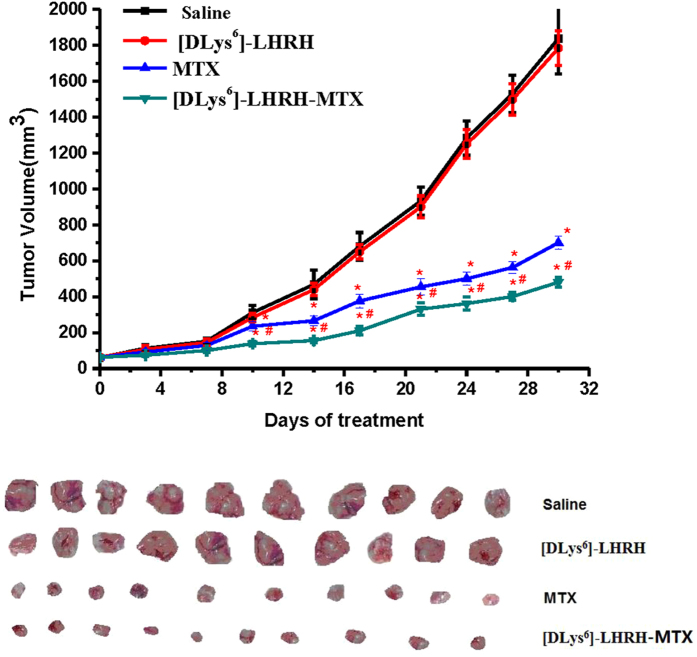
Anti-tumor activity of saline, [DLys^6^]-LHRH, MTX and [DLys^6^]-LHRH-MTX on PC-3 tumor-bearing mouse model. The controls were administeredsaline. Tumor volume were recorded and the tumors were harvested at the end of experiments (e.g. 30 days). (n = 10, *p < 0.05 vs. saline, ^#^p < 0.05 vs. MTX).

The *in vivo* anti-tumor activity of the therapeutic agents were assessed on mice bearing PC-3 tumor xenografts. At the end of the study, the mice were sacrificed and the whole tumor tissues were obtained. Both MTX and [DLys^6^]-LHRH-MTX exhibited significant anti-tumor effects. Whereas treatment with [DLys^6^]-LHRH alone did not affect the tumor volume. As shown in Fig. 5 and Table. 2, the mean tumor volume after [DLys^6^]-LHRH-MTX treatment was 480.17 ± 15.74 mm^3^, which was much smaller in comparison to the other groups (p < 0.05). The inhibition ratio of tumor volume in the [DLys^6^]-LHRH-MTX treated group was approximately 73.87%, which was significantly higher than that of the MTX treated group (61.99%, p < 0.05) and the [DLys^6^]-LHRH treated group (2.88%, p < 0.01), suggesting the highest therapeutic activity was derived from [DLys^6^]-LHRH-MTX.

**Figure 6:**
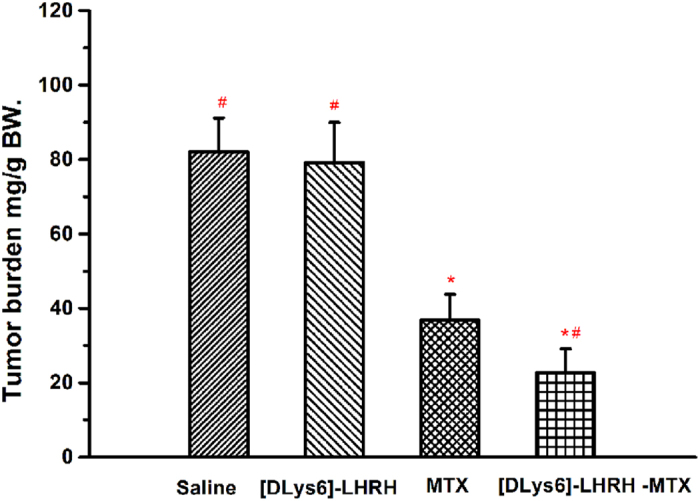
Tumor burden from mice treated with saline, [DLys^6]^-LHRH, MTX or [DLys^6^]-LHRH -MTX at day 30 post-tumor cell inoculation. (n = 10, *p < 0.05 vs. saline, ^#^p < 0.05 vs. MTX).

In addition, as shown in Table 2, the mean weight the of tumor tissues in the [DLys^6^]-LHRH-MTX treated group was significantly less than that of the saline control group (more than 3-fold less, p < 0.01), the [DLys^6^]-LHRH group treated group (3-fold less, p < 0.01) and MTX group (approximately 1-fold less, p < 0.05), indicating that LHRH modification further increased the therapeutic efficacy of MTX^22^,^30^,^31^. As shown in Fig. 6 the tumor burden was decreased from 81.62 ± 17.12 mg/g body weight in tumor-bearing control animals to 22.65 ± 6.18 mg/g body weight, in animals treated with [DLys^6^]-LHRH-MTX (P<0.05 compared with saline control, MTX and [DLys^6^]-LHRH-saline treated animals). Tumor burden was unchanged in the treatment groups receiving either [DLys^6^]-LHRH alone or the saline control (Fig. 6). Tumor volume doubling time was also significantly extended from 6.13 days in the control animals to 9.67 days in the mice treated with [DLys^6^]-LHRH-MTX (Table 2). These results confirm that [DLys^6^]-LHRH-MTX is indeed more effective than MTX alone *in vivo*. This efficacy could be attributed to the targeting features of [DLys^6^]-LHRH-MTX that allow for better localization of treatment doses in tumor tissue and cells compared with the non-targeted treatments.

**Figure 7:**
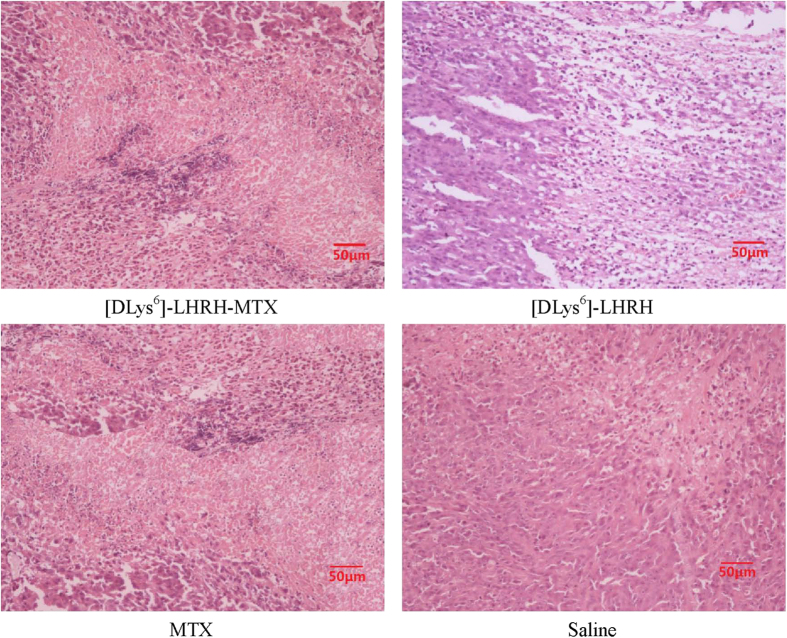
Hematoxylin/eosin staining of tumor sections of PC-3 xenografts, Animals treated with saline showed predominantly viable cells,whereas tumors from animals treated with.

Histologically, the tumor tissue of conjugate-treated animals was found to consist mainly of necrotic cells and fluid. Hematoxylin/eosin stained tumor sections of PC-3 xenografts treated with saline or [DLys^6^]-LHRH alone showed predominantly viable cells (Fig. 7), whereas tumors from animals treated with [DLys^6^]-LHRH-MTX showed a high degree of necrosis, when compared to animals treated with the unconjugated lytic peptide and LHRH (Fig. 7). The untreated tumors consisted of sheets of cells with large vesicular hyperchromatic nuclei and prominent nucleoli; many mitotic figures were also seen. In most instances, the tumor margin was rich in blood vessels, which penetrated the rim of the neoplastic tissue. In contrast, the tumors of the LHRH–MTX treated mice were pale and poorly vascularized, although some intact vessels were present within the tissue.

## Conclusion

In the study, targeted drug [DLys^6^]-LHRH-MTX was successfully synthesized and characterized. Upon evaluated in human prostate cancer prostate cancer cell lines (PC-3, DU145 and LNCaP), this targeted drug delivery system displayed a significantly enhanced induction of apoptosis and the inhibition of cell growth activity compared to the non-targeted conjugates. The superior anti-tumor efficacy of [DLys^6^]-LHRH-MTX was further proveddemonstrated in a PC-3 tumor-bearing mouse model. Our data demonstrate that [DLys^6^]-LHRH-MTX provides an improved therapeutic index for MTX and may be useful for treatment of LHRH receptor-positive cancers.

## Material and Methods

### Material

MTX was purchased from Jiangsu Hengrui Medicine Co. Ltd. (Jiangsu, China). LHRH was purchased from Shanghai GL Biochem Co. Ltd. (Shanghai, China). EDC, N-hydroxy succinimide(NHS) and 4-dimethylaminopyridine were all purchased from Sigma (Steinheim, Germany), Dimethyl sulfoxide(DMSO) and N,N-Dimethylformamide(DMF), Triethylamine(TEA) were all purchased from Merck(Darmstadt, Germany). All other chemicals and reagents used were of analytical grade.

### Synthesis

#### Synthesis of MTX-active ester

The synthesis^22^ of [DLys^6^]-LHRH-MTX and its proposed mechanism of action are shown in the supporting information Fig1. S1. Initially, an MTX-active ester was formed and then conjugated to [DLys^6^]-LHRH. MTX was activated with NHS as described previously. Under dry conditions, MTX (676.32 mg, 1.49 mmol) was dissolved in DMF (10 mL) and reacted with NHS (514.45 mg, 4.47 mmol), DMAP(89.37 mg, 0.73 mmol) and EDC.HCL (869.33 mg, 4.48 mmol) for 2 h at room temperature and then for 18 h at 4 °C in the dark. The precipitate of the reaction product was removed by centrifugation, and the supernatant containing the active ester derivative was concentrated under vacuum at 37 °C. The compound was purified by silica gel column chromatography with a 85.16% yield. ^1^HNMR (400 MHz, δ(ppm) in CDCl_3_): 2.19(t, 2H, CH_2_), 2.33(d, 2H, CH_2_), 2.85(d, 4H, CH_2_), 2.93(s, 3H, CH_3_), 4.08 (s, 4H, NH_2_), 4.47 (m, 1H, CH), 4.77(s, 2H, CH_2_), 6.84(d, 2H, CH), 7.84(d, 2H, CH), 8.12 (d, H, NH), 8.67(s, 1H, CH), 11.21(s, 1H, OH).

### Synthesis of [DLys^6^]-LHRH –MTX

The conjugate^22^,^32^ was prepared by incubating the active ester of MTX with [DLys^6^]-LHRH in PBS, pH 8.2 at a molar ratio of MTX to [DLys^6^]-LHRH of 1.2:1 and stirring at 4 °C overnight. The MTX- conjugate was dialyzed against PBS (pH 8.5) overnight. The compound was purified by preparative high-performance liquid chromatography with a 92.78% yield. The conjugate was analyzed by LC-MS as described below and was subsequently stored at −20 °C. ESI-MS([M+2H]^2+^), calculated for C_77_H_101_N_25_O_16_ 1632.78, found1632.58 (Fig. S1). The mass spectrum of LHRH-MTX is shown in in the supporting information Fig. S1.

### Cell culuture

#### Prostate cancer cell lines

(PC-3, DU145 and LNCaP) (LHRH receptor over-expressing cancer cell line) and HPrF (non-LHRH receptor expressing cancer cell line) were purchased from the American Type Culture Collection (Manassas, VA). **PC-3 cells and HPrF cells** were maintained in T-media (Gibco Invitrogen, CA) supplemented with 10% fetal bovine serum (FBS) (Gibco Invitrogen, CA) and 1% penicillin/streptomycin (Cellgro Mediatech, nc., VA). The **LNCaP** cells were grown in RPMI 1640 medium(Gibco Invitrogen, CA) containing 10% FBS and 1% penicillin/streptomycin and the **DU145** cells were grown in Eagle’s minimum essential medium (MEM) (Gibco Invitrogen, CA) with 2 mM L-glutamine and Earle’s BSS, 1.5 g/L sodium bicarbonate, 0.1mM non-essential amino acids, 1 mM sodium pyruvate, 10% FBS and 1% penicillin/streptomycin. The cultures were maintained at sub-cofluency at 37 °C in a humidified atmosphere of 5% CO_2_/95% air. All experiments were performed on cells in the logarithmic growth phase.

### Cytotoxicity assay

The cytotoxic effect of MTX, [Dlys^6^]-LHRH, and [Dlys^6^]-LHRH-MTX was evaluated using the methylthiazol tetrazolium (MTT) assay (Sigma Aldrich, MO), which determines the number of viable cells from the formazan crystals produced by metabolic activity^26^,^33^,^34^. The cells were plated in 96-well plates at 5 × 10^3^ cells/well in triplicate, and were allowed to reattach overnight. Culture media with various concentrations of MTX or [Dlys^6^]-LHRH or [Dlys^6^]-LHRH-MTX were then added in triplicate, and the incubation was continued for an additional 24 h. After removing the supernatant, DMSO was added (150 μL per well) to dissolve the formazane of MTT. The absorption at 570 nm was recorded with an ELISA plate reader (Bio-Rad, Microplate Reader 550 and the growth inhibitory (GI) rate was calculated according to the following equation: GI(%) = 100 − [T − B)/(C − B) × 100].

In the equation, T represents the absorption value of the treatment group; C is the absorption value of the control (untreated) group; and B refers to the absorption value of the culture medium. IC_50_ values (μg/mL) were calculated with SPSS software. In a competition assay, Prostate cancer cells (5,000 per well) were separately incubated in 96-well plates in triplicate with [DLys^6^]-LHRH–MTX alone and [DLys^6^]-LHRH free peptide and [DLys^6^]-LHRH–MTX together, and the cells were incubated for 24 hr. The plates were then analyzed with the MTT method.

### Apoptosis analysis with Annexin V-FITC/PI staining

To quantitatively evaluate the induction of apoptosis by flow cytometry, Annexin-V-FITC/PI staining was performed^35^. Briefly, PC-3 cells were seeded in a 6-well culture plate at a density of 2 × 10^5^ cells per well, and treated with various concentrations of MTX or [Dlys^6^]-LHRH or [Dlys^6^]-LHRH-MTX for 24 h. The cells were harvested and then stained with Annexin V-FITC/PI solution (1:1, v/v, 100 ml) in the dark for 15 min at room temperature. After washing with PBS, the cell suspensions in buffer were gently vortexed, and analyzed within 1 h by flow cytometry (Beckton Dickinson, CA, Costa Rica).

### *In vivo* study, Animals

Four week-old male athymic BALB/c nude mice were obtained from Sino-British SIPPR/BK Laboratory Animal Ltd (Shanghai, China). The animals were housed in sterile cages under laminar flow hoods in a temperature-controlled room with a 12h light/12h dark schedule and were fed autoclaved chow and water ad libitum.

### Ethics statement

All animal work were conducted under the approved guidelines of the Shanghai Institute of Planned Parenthood Research (Shanghai, China) and approved by the Animal Care Committee of the Shanghai Institute of Planned Parenthood Research (Shanghai, China) and were performed in accordance with the approved guidelines.

### Subcutaneous implantation of PC-3 cells

PC-3 cells were harvested from subconfluent cultures after a brief exposure to 0.25% trypsin and 0.2% EDTA. Trypsinization was stopped by adding medium containing 10% FBS. The cells were washed once in serum-free medium and resuspended in PBS. Only suspensions consisting of single cells with >90% viability were used for the injections. PC-3 cells (1 × 10^7^) in 100 μL PBS suspended in Matrigel (0.1 mg, Collaborative Biomedical Products Becton Dickinson Labware, Bedford, MA) were injected subcutaneously into the interscapular area using a 27-gauge needle^32^.

### Experimental protocol

On day 21, after PC-3 cell implantation, mice bearing tumors between 100 and 150 mm^3^ were randomized into the following four treatment groups (n = 10): saline controls, MTX treatment, [Dlys^6^]-LHRH-MTX treatment and MTX plus [Dlys^6^]-LHRH treatment. Treatments were made in 100 μL PBS and were given intravenously by tail vein injection twice weekly for 4 weeks. The vehicle-treated group received an equivalent amount of PBS. Tumor volumes were measured with calipers and the body weights of the mice were measured twice a week. Tumor volumes were calculated with the following formula: V(mm^3^) = length × width^2^/2, where width represents the shortest measurement in millimeters. The animals were euthanized 7 days after the last treatment. Body weights, tumor weights and organ weights were recorded.

### Statistical analysis

Data were presented as Mean ± SD. Statistical analyses were done by using SPSS 20.0 software. Differences were determined by ANOVA and Student’s t test when appropriate. The *p* value of <0.05 was considered significant and the *p* value of <0.01 was considered highly significant.

## Additional Information

**How to cite this article**: Zhu, S. *et al*. A conjugate of methotrexate and an analog of luteinizing hormone releasing hormone shows increased efficacy against prostate cancer. *Sci. Rep.* **6**, 33894; doi: 10.1038/srep33894 (2016).

## Supplementary Material

Supplementary Information

## Figures and Tables

**Table 1:** The IC_50_ (μmol/L) values of [DLys^6^]-LHRH-MTX, MTX and [DLys^6^]-LHRH against different cells (means ±  SD, n = 3).

Groups	MTX (μmol/L)	[DLys^6^]-LHRH-MTX (μmol/L)	**[DLys**^**6**^**]-LHRH** **(μmol/L)**
PC-3	6.34 ± 1.01^c^,^d^	1.02 ± 0.18^a^,^d^	n.d
DU145	8.03 ± 1.29^b^,^d^	1.53±0.27^a^,^d^	n.d
LNCaP	9.68 ± 1.24^b^,^c^	1.93 ± 0.19^a^,^b^,^c^	n.d
HPrF	42.33 ± 7.25	110.77 ± 15.31	n.d

(n.d. = not determined,

^a^p < 0.05, vs. MTX,

^b^p < 0.05, vs. PC-3,

^c^p < 0.05, vs. DU145,

^d^p < 0.05, vs. LNCaP).

**Table 2:** The mean tumor volume and weight after treatments (means ±  SD, n = 10).

Treatment	Mean tumor volume (mm3)		Inhibition ratio of tumor volume (%)	Tumor volume doubling time (days)	Mean tumor weight (g)	Inhibition ratio of tumor weight (%)
	Initial	Final				
Saline	61.28 ± 14.86	1837.49 ± 197.33	/	6.13	2.04 ± 0.39	/
[DLys^6^]-LHRH	62.49 ± 13.70	1784.58 ± 95.99	2.88	6.13	1.95 ± 0.34	4.41
MTX	63.38 ± 7.38	700.39 ± 36.48^a^	61.88	8.88	0.82 ± 0.12^a^	59.90
[DLys^6^]-LHRH-MTX	62.77 ± 8.99	480.17 ± 15.74^a^,^b^	73.87	9.67	0.61 ± 0.16^a^	70.10

^(a^p < 0.05, vs. Saline;

^b^p < 0.05, [DLys^6^]-LHRH-MTX vs. MTX).
