# Supplementary material for: A conjugate of methotrexate and an analog of luteinizing hormone releasing hormone shows increased efficacy against prostate cancer
Source: Sci Rep. 2016 Sep 22;6:33894. doi: 10.1038/srep33894 (PMC5032167; doi:10.1038/srep33894)
Supplement: Supplementary Information [file srep33894-s1.pdf]

A conjugate of methotrexate and an analog of luteinizing hormone releasing hormone shows increased efficacy against prostate cancer

Shengsheng Zhu, Qinxia Wang, Juan Jiang, Yongwei Luo, Zuyue Sun*

Department of Pharmacology and Toxicology, Shanghai Institute of Planned Parenthood Research, Shanghai, 200032, PR China

* Corresponding author. Tel.: +86 21 64229909; fax: +86 21 64043044.
E-mail address: sunzy64@163.com (ZY. Sun).

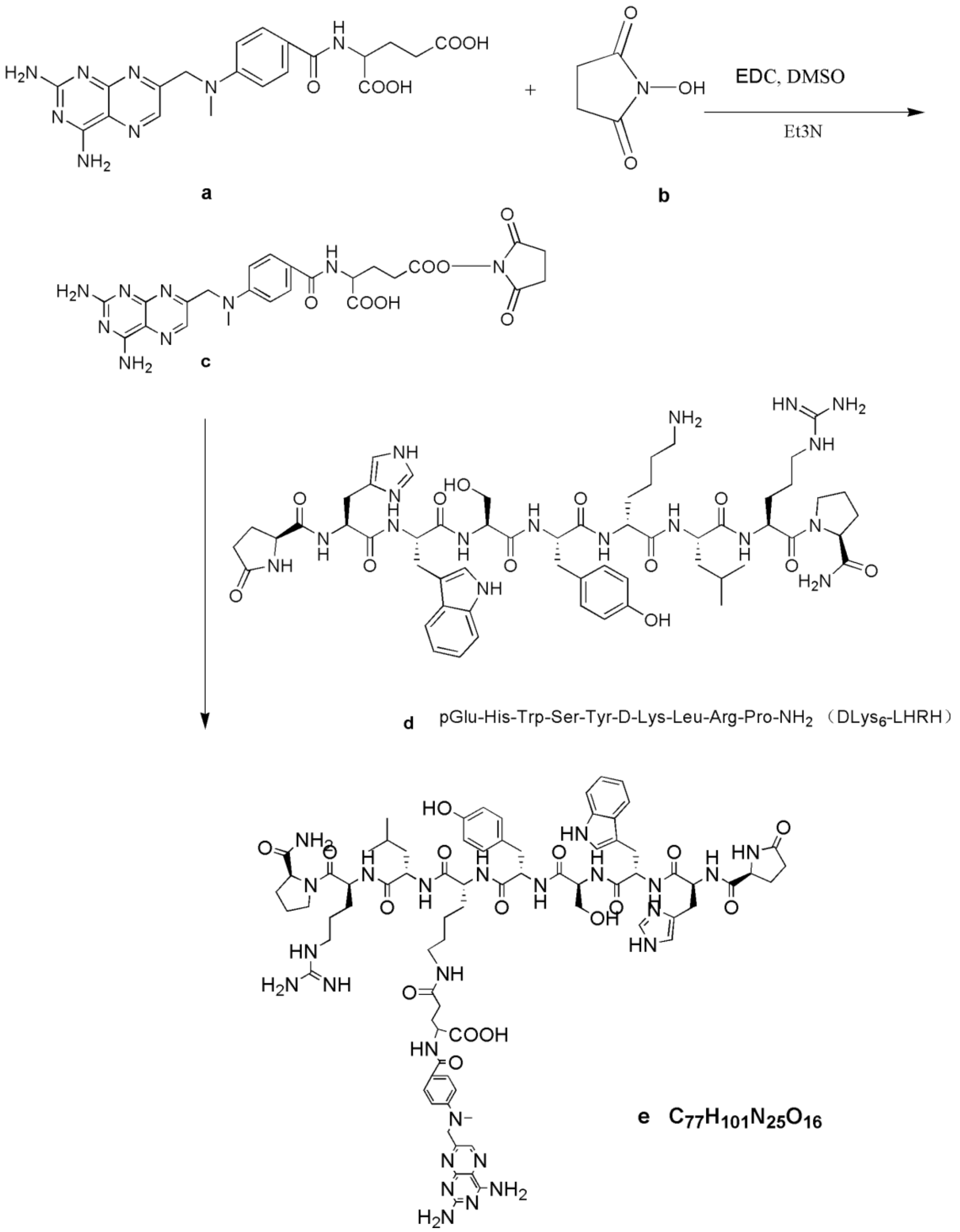

Fig1. S1. Synthetic route for [DLys^{6}]-LHRH-MTX

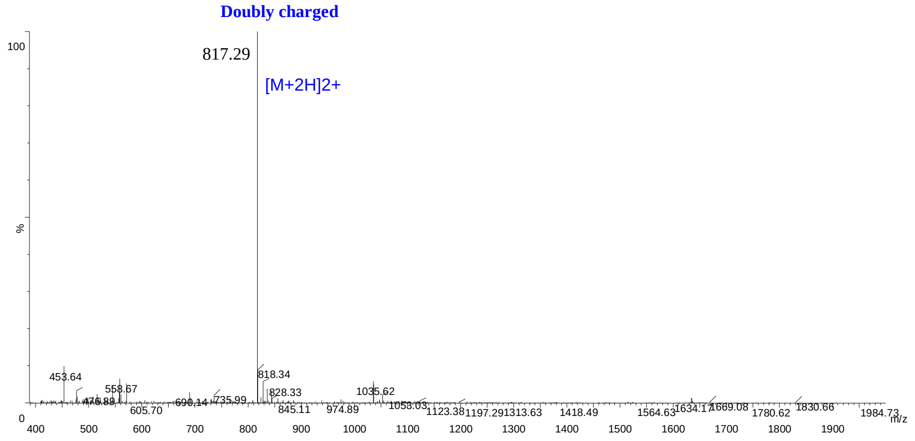

Fig2. S1. Mass spectra of [DLys^{6}]-LHRH-MTX
